# The Influence of the Ar/N_2_ Ratio During Reactive Magnetron Sputtering of TiN Electrodes on the Resistive Switching Behavior of MIM Devices

**DOI:** 10.3390/ma18173940

**Published:** 2025-08-22

**Authors:** Piotr Jeżak, Aleksandra Seweryn, Marcin Klepka, Robert Mroczyński

**Affiliations:** 1 Warsaw University of Technology, Institute of Microelectronics and Optoelectronics, Koszykowa 75, 00-662 Warsaw, Poland; 2 Warsaw University of Technology, Center of Advanced Materials and Technology CEZAMAT, Poleczki 19, 02-822 Warsaw, Poland; 3 Institute of Physics, Polish Academy of Sciences, Aleja Lotników 32/46, 02-668 Warsaw, Poland

**Keywords:** resistive switching, RRAM, reactive magnetron sputtering, electronic applications

## Abstract

Resistive switching (RS) phenomena are nowadays one of the most studied topics in the area of microelectronics. It can be observed in Metal–Insulator–Metal (MIM) structures that are the basis of resistive switching random-access memories (RRAMs). In the case of commercial use of RRAMs, it is beneficial that the applied materials would have to be compatible with Complementary Metal-Oxide-Semiconductor (CMOS) technology. Fabricating methods of these materials can determine their stoichiometry and structural composition, which can have a detrimental impact on the electrical performance of manufactured devices. In this study, we present the influence of the Ar/N_2_ ratio during reactive magnetron sputtering of titanium nitride (TiN) electrodes on the resistive switching behavior of MIM devices. We used silicon oxide (SiOx) as a dielectric layer, which was characterized by the same properties in all fabricated MIM structures. The composition of TiN thin layers was controlled by tuning the Ar/N_2_ ratio during the deposition process. The fabricated conductive materials were characterized in terms of chemical and structural properties employing X-ray photoelectron spectroscopy (XPS) and X-ray diffraction (XRD) analysis. Structural characterization revealed that increasing the Ar content during the reactive sputtering process affects the crystallite size of the deposited TiN layer. The resulting crystallite sizes ranged from 8 Å to 757.4 Å. The I-V measurements of fabricated devices revealed that tuning the Ar/N_2_ ratio during the deposition of TiN electrodes affects the RS behavior. Our work shows the importance of controlling the stoichiometry and structural parameters of electrodes on resistive switching phenomena.

## 1. Introduction

In recent years, there has been an increase in data-computing systems such as artificial intelligence, machine learning, and big data analysis. All of these applications take advantage of memories that are used to store digital data [1]. Depending on the function that memory serves in a system, it can be classified as volatile or non-volatile. The majority of the memory market is made up of static random-access memory (SRAM) and dynamic random-access memory (DRAM) as volatile memories, and Flash as non-volatile memories [2]. SRAMs and DRAMs perform at high speed but have low capacity, while Flash memories can store more data but have longer write and read times [1,2]. Regardless of the maturity of these technologies, they are expected to be unable to fulfill the requirements of emerging computing applications, which are faster speed, higher capacity, and lower power consumption. For this reason, new types of memories are being developed, which are named emerging memories [1,2,3].

Among these newer technologies, the following can be mentioned: magnetic random-access memory (MRAM), phase-change memory (PCM), ferroelectric random-access memory (FeRAM), and resistive random-access memory (RRAM) [3,4]. Special attention deserves RRAMs due to their simple memory cell structure, high endurance, high speed, and scalability [5]. In these devices, the state of a cell is indicated by one of the resistance levels, which are a high-resistive state (HRS) and a low-resistive state (LRS) that are cyclically changed depending on applied voltage [6].

A single memory cell of the RRAM device consists of a MIM structure [5]. In these devices, resistive switching is driven by the migration of ions within the dielectric layer of a MIM structure. These migrations can occur either in transition-metal oxides, such as TiOx, HfOx, or TaOx, or in solid electrolytes. In transition-metal oxides, resistive switching is typically caused by the migration of oxygen anions toward the anode under an applied voltage. This process leads to the formation of conductive paths composed of oxygen vacancies, which enable current flow. In RRAM devices that use solid electrolytes as the dielectric layer, the conductive path is formed by the migration of cations from one of the electrodes through the dielectric. Specifically, cations from an electrochemically active electrode—such as Ag or Cu—drift through the electrolyte under an electric field, thereby creating the conductive path between the electrodes. Additionally, interfacial resistive switching has been reported in certain perovskite materials, attributed either to charge trapping or to electrochemical reactions [7]. However, RRAMs based on oxygen anion migration exhibit the highest endurance and retention, making them the most promising candidates for non-volatile memory applications [3]. For the remainder of this work, RRAM devices based on oxygen anion migration will be referred to simply as RRAM.

Electrode materials that are most commonly used to fabricate RRAMs are Pt, Au, Ag, Ni, Ti, and Cu, but back-end-of-line (BEOL) manufacturing conditions demand low-temperature processing and industry-friendly materials like TiN or TaN. Even though resistive switching is obtained in a dielectric layer, the electrode materials used to fabricate RRAM significantly impact the device’s electrical performance. For example, the unipolar switching mode is most commonly observed when both the bottom electrode (BE) and top electrode (TE) are fabricated of noble materials like Pt or Ru. However, changing one of these electrodes with oxidizable material like TiN or Ti will result in bipolar resistive switching [8].

Due to its compatibility with CMOS technology, titanium nitride is commonly used in the semiconductor industry as a gate material in MOSFET transistors. More than that, titanium nitride can be fabricated with a Physical Vapor Deposition (PVD) process like reactive magnetron sputtering, which is a low-temperature process [9]. TiN can be used to fabricate MIM structures under BEOL conditions, which is a crucial aspect, because RRAMs are expected to be fabricated on the metal layers of a CMOS chip [8]. All of these aspects make titanium nitride an ideal candidate for fabricating RRAM devices. Information about RRAM devices with TiN as electrode material can be found in the literature, where various resistive switching behaviors are observed [10]. This is due to the fact that electrodes were fabricated with different deposition conditions, which resulted in different properties [10]. However, the application of the same deposition method does not guarantee the fabrication of layers with the same parameters, while the deposition process can be optimized to obtain the desired results [9]. Sun et al. [10] showed that different switching modes can be determined by tuning the crystallinity state of the TiN electrode deposited in magnetron sputtering, where a pure TiN target was used.

In this work, we present the results of tuning the Ar/N_2_ ratio during the deposition of TiN electrodes of the MIM structures on resistive switching behavior. As reported in our previous works exhibiting resistive switching properties, silicon oxide (SiO_x_) was used as a dielectric layer [11,12]. The fabricated conductive and dielectric films were deposited at room temperature in the DC-pulsed reactive magnetron sputtering process. The technology of deposited titanium nitride layers was validated with spectroscopic ellipsometry measurements. The stoichiometry and crystallinity of TiN thin films were controlled by tuning the Ar content in the reactive gases flow. Predictions of TiN thin films’ stoichiometry based on spectroscopic ellipsometry were confirmed and more precisely defined by XPS measurements. This work highlights the significance of chemical and structural control of the electrode material employed in MIM devices on the RS mechanism behavior. The presented results are essential for the future optimization of RRAM devices.

## 2. Materials and Methods

Reactive magnetron sputtering allows for the deposition of compound films through the bombardment of a target material by ionized sputtering gas atoms, accompanied by the introduction of reactive gas species into the vacuum chamber. To fabricate TiN layers, the pure titanium target with a purity of 99.99%, argon, and nitrogen, both with a purity of 99.999999%, were used as sputtering and reactive gas, respectively. Argon content in the reactive gases flow was tuned from 20 sccm (standard cubic centimeters per minute) to 60 sccm with a 10 sccm step. A lower Ar flow, such as 10 sccm, could further decrease the nitrogen content and potentially improve crystallinity or stoichiometry. However, in our experimental setup, Ar flows below 20 sccm resulted in unstable plasma and reduced sputtering efficiency, making producing uniform and reproducible films difficult. For this reason, we selected 20–60 sccm as a safe and effective operating window. Nitrogen content was set to 7 sccm. The remaining process parameters were as follows: power applied to the reactive chamber was 1 kW, pressure was 1 mTorr, and the deposition time was 2 min. The decision to vary only the Ar flow while keeping the N_2_ flow constant at 7 sccm was intentional and based on the previous results and the need to simplify the parameter space while maintaining process stability. By fixing the N_2_ flow, we ensured a constant reactive environment and minimized fluctuations in nitrogen incorporation due to gas flow instability. Meanwhile, adjusting the Ar flow primarily modulates the sputtering power and kinetic energy of the arriving Ti species, thus influencing the Ti/N ratio indirectly. This approach is supported by prior reports [9], which show that varying the inert gas flow during reactive sputtering affects the target poisoning and film stoichiometry through changes in plasma density and target utilization efficiency. This design allowed us to assess the effect of Ti enrichment vs. nitrogen incorporation in a controlled manner and observe the evolution of film properties (optical, chemical, structural, and electrical) with sufficient resolution. The deposited TiN layers were characterized by spectroscopic ellipsometry (Horiba Jobyn-Yvon UVISEL, Horiba, Lille, France), where the real part of the relative permittivity of the deposited layers was obtained. By controlling at which wavelength λ, the real part of relative permittivity Re(ε) is equal to 0, it is possible to determine if the deposited film has more or less titanium content in TiN composition as reported in [13]. It has already been shown that the stoichiometric titanium nitride is characterized by the real part of the relative permittivity equal to 0 at λ = 480 nm. The nitrogen-to-titanium ratio in the TiN layer can be inferred from the zero-crossing wavelength of the real part of the relative permittivity. A zero-crossing at shorter wavelengths indicates a titanium-rich composition, while a shift to longer wavelengths suggests increased nitrogen content [13]. Based on this method, three samples of TiN thin films were selected for further chemical and structural characterization and fabrication of MIM devices. One with more Ti content, one nearly stoichiometric, and one with more N content.

MIM structures were fabricated on 2-inch silicon (Si) substrates. Before processing, the surface of Si wafers was cleaned in 40:1 hydrofluoric acid (HF) with deionized (DI) water. The processing sequence of MIM devices was as follows: the first step was standard UV photolithography employed with negative-tone photoresist (NLOF 2070, MicroChemicals GmbH, Ulm, Germany) in order to structurize the TiN BE using the lift-off procedure. The sequence was repeated in the case of the dielectric layer and TE. The exact chemical composition characterized the bottom and top electrodes, depending on the type of MIM device, i.e., with more Ti content, nearly stochiometric, or with an excess of N content. In each case, SiO_x_ was used as an insulator layer that was deposited in the same sputtering process. Silicon oxide was fabricated at a power of 200 W, pressure of 3 mTorr, 20 sccm of O_2_, and 30 sccm of Ar. The dielectric layer was deposited for 4 min and 41 s. All films were deposited at room temperature (25 °C) using a DC-pulsed reactive magnetron sputtering process. While TiN deposition is often performed at elevated substrate temperatures to promote larger crystallite sizes, improved crystallinity, and a reduced lattice constant, our approach was intentionally chosen to ensure compatibility with BEOL CMOS integration, where thermal budgets are strictly constrained. For instance, Mustapha and Fekkai [14] demonstrated that increasing the substrate temperature from 300 °C to 500 °C led to a measurable increase in crystallite size. By depositing at room temperature, we prioritized process compatibility with BEOL CMOS integration. Oxford Instruments Plasmalab System 400 (Bristol, England) was used for all examined materials’ reactive DC-pulsed magnetron sputtering process.

**Figure 1:**
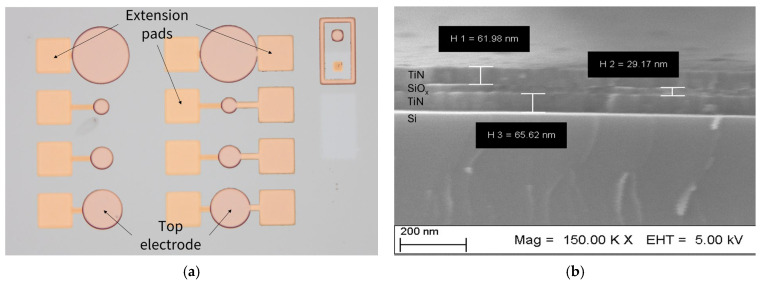
(**a**) Matrix of fabricated MIM structures; (**b**) cross-section image of fabricated MIM structure (SEM).

Test MIM structures were fabricated on silicon substrates as matrices. In each matrix, MIM structures were formed with extension pads to only BE, and the remaining structures with extension pads to both BE and TE, as shown in Figure 1a. The cross-sectional image of a single cell is shown in Figure 1b. The electrical measurements were conducted with the Keysight B1500A (Keysight, Santa Rosa, CA, USA) semiconductor characterization system equipped with the SUSS-PM8 probe station. The voltage sweep was performed in quasi-static mode with a rate of 0.1 V/s. The results of electrical measurements in this work refer to the structures with a diameter of 160 µm.

XRD measurements were performed using the Panalytical X’Pert Pro MRD diffractometer (Panalytical, Almelo, The Netherlands) with an X-ray tube generating radiation at a wavelength of 1.54056 Å and a hybrid two-bounce Ge (220) monochromator, and a Pixel detector as described in [15]. Measurements were performed in incidence X-ray diffraction (GIXRD) geometries [16]. The lattice constants were calculated using the Nelson–Riley extrapolation method [17].

(1) $$ALP\sim \frac{1}{2}\left(\frac{\cos^{2}\theta }{sin\theta }+\frac{\cos^{2}\theta }{\theta }\right)$$

The crystallite sizes (D) weighted average was estimated using the Sherrer formula, calculated for individual diffraction reflexes and taking into account their maximal intensity in the obtained diffractogram.

XPS measurements were performed using a Prevac system with a Scienta R4000 hemispherical analyzer with a pass energy of 200 eV (Scienta Omicron, Uppsala, Sweden). The data were collected using the monochromatic Al K_α_ (1486.7 eV) excitation, working with a power of 150 W [18]. The C 1s line was set at 285.0 eV to calibrate the energy scale. Samples were measured as received. The commercial CASA XPS software package (Casa Software Ltd., Teignmouth, UK, version 2.3.25PR1.0) was used to analyze the spectra [19]. The spectra were fitted with a Shirley background and deconvoluted with a mixed Gaussian–Lorenzian function, except for TiN, which was fitted with an asymmetric function as reported in [20].

## 3. Results and Discussion

### 3.1. Characterization of TiN Thin Films

**Figure 2:**
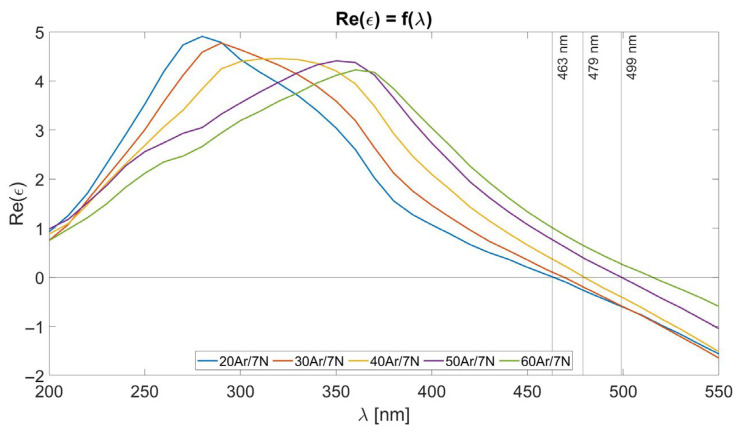
The real part of the relative permittivity of deposited TiN layers after spectroscopic ellipsometry.

As described in Section 2, titanium nitride layers were deposited in a DC-pulsed reactive magnetron sputtering process at various conditions and characterized with spectroscopic ellipsometry. The results of optical measurements are shown in Figure 2.

Each deposited film was characterized by a real part of relative permittivity equal to 0 at different wavelengths. One of the investigated titanium nitride layers exhibited a zero-crossing of the real part of the relative permittivity (Re(ε) = 0) at λ = 479 nm, indicating that the film is nearly stoichiometric TiN. Two of the fabricated titanium nitride layers showed zero-crossing at shorter wavelengths, which suggests a higher titanium content. In contrast, two other layers exhibited this parameter at longer wavelengths, implying a higher nitrogen content in the TiN composition. For further analysis, three types of TiN layers were selected, which were named TiN-1, TiN-2, and TiN-3, with Re(ε) = 0 occurring at λ = 499 nm, λ = 479 nm, and λ = 463 nm, respectively, as shown in Figure 2. The remaining two samples had intermediate properties and were excluded to maintain focus and avoid redundancy. Selected layers were deposited in accordance with the Ar/N_2_ ratio as shown in Table 1.

X-ray photoelectron spectroscopy (XPS) was employed to analyze the chemical composition of the investigated TiN films. The survey scan revealed the presence of titanium (Ti), nitrogen (N), oxygen (O), and carbon (C). High-resolution scans were performed for the Ti 2p, N 1s, O 1s, and C 1s core-level lines. The atomic percentage content is presented in Table 1.

The detected C 1s signal originates from surface carbon contamination, which was not presputtered with Ar^+^ prior to the XPS measurements, as the surface had not been cleaned during the manufacturing of the MIM structures. Cleaning the TiN samples’ surface would alter their chemical contamination, resulting in inconsistency with the layers used for fabricating the devices analyzed in this study. Since the electrical behavior of the devices is influenced by the actual (possibly contaminated) surface of the electrodes, we felt it was important to report the chemical state as-fabricated. Nevertheless, the XPS measurements enabled determination of the dominant chemical properties of the sample without interfering with its composition and structural parameters, which would certainly be affected by ion sputtering.

**Figure 3:**
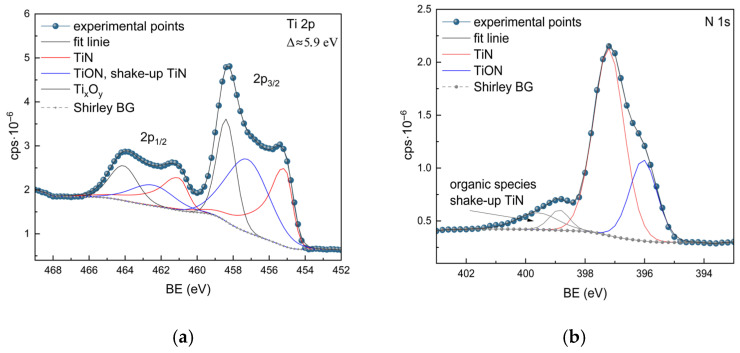
The XPS spectra of Ti 2p (**a**) and N 1s (**b**) from the TiN-3 sample (as a reference).

The individual core-level spectra of each element were deconvoluted by fitting components associated with distinct chemical states. The components of each element’s spectral lines are qualitatively compatible across all samples. A detailed description of the components for the TiN-3 sample is presented in Figure 3. The Ti 2p line exhibits spin-orbit splitting, with a binding energy difference of about 5.7 to 6.0 eV [21]. Three components have been identified with BE close to 455 eV, 457 eV, and 458 eV, corresponding to TiN, TiON, and Ti_x_O_y_, respectively [20,22]. The component with the BE close to 457 eV, attributed to TiON, partially overlaps with the TiN shake-up component [22].

The N 1s line has been deconvoluted into two components, attributed to TiN (BE of 397 eV) and TiON (BE of 396 eV). The remaining components with higher binding energies likely correspond to organic species and overlap with the TiN shake-up line [20]. The presence of carbon is likely due to adventitious carbon contamination, which is typical for surfaces exposed to air. The presence of oxygen in TiN films deposited by the magnetron sputtering method is well known [23].

As shown in Table 1, decreasing the argon content in the reactive gases flow caused a decrease in nitrogen in the chemical composition of deposited TiN layers. The seeming trend arises from the complex dynamics of reactive sputtering. While reducing Ar content, which increases the relative proportion of N_2_ in the gas mixture, it also reduces the sputtering yield of Ti due to lower ion bombardment energy. Simultaneously, the surface of the Ti target may become increasingly poisoned with TiN, reducing the effective sputtering rate of Ti atoms. In this case, the increased nitrogen content in the plasma does not translate directly to higher nitrogen incorporation in the film because of lower titanium flux and reduced adatom energy. As a result, lower Ar flows can paradoxically lead to Ti-rich films due to reduced reactivity and plasma density. This non-linear behavior has been observed in previous literature [23].

**Figure 4:**
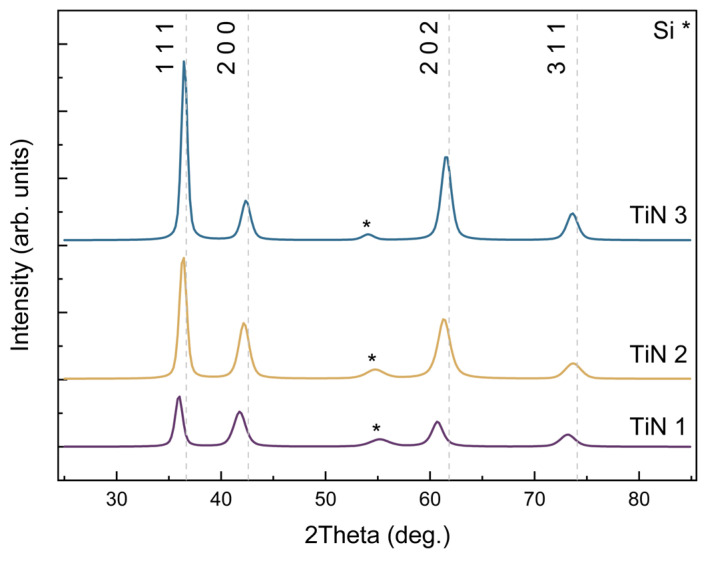
Results of the XRD measurements of the investigated TiN films.

After XRD measurements, the crystallographic structures of deposited TiN films were revealed. Identified diffraction peaks are in accordance with peaks described in the crystallography base (ref. code 00-0-38-1420), which refers to TiN cubic structure with Fm-3m space group with number 225. The given reference lattice constant is a = 4.2417 Å. During analysis, four peaks were observed, as shown in Figure 4. The results of the measurements indicate significant differences in the crystal quality of TiN layers, which are dependent on process parameters.

The relatively low intensity of diffraction peaks and high full width at half maximum (FWHM), all of the maxima are shifted towards lower angles, which indicates small crystallite sizes. The calculated weighted average crystallite size D is 0.8 nm. That suggests that the TiN-1 layer is nanocrystalline.

Recorded for TiN-2 layer, higher intensity and smaller FWHM of diffraction peaks indicate a higher crystalline order of atoms in the lattice structure. The lower lattice constant a = 4.256 Å, and at the same time, closer to the reference value, denotes an improvement in the crystalline structure of the material. This is also confirmed by the significantly higher weighted average crystallite size D = 46.43 nm. The shift of diffraction peaks toward the reference values decreased, confirming for sample TiN-2 what could be consistent with a larger crystallite size.

In the case of the TiN-3 layer, the highest intensity of diffraction peaks and the lowest FWHM were noticed. Again, a significant increase in weighted average crystallite size was observed (D = 75.74 nm). The increase in lattice constant of layer TiN-3, a = 4.262 Å, compared to the value a = 4.256 Å of layer TiN-2, falls within the error margins of linear approximation applied to determine the apparent lattice constant in the materials. The higher lattice constant may suggest the presence of interstitial atoms or tensile stress. The evident inconsistency arises from a combination of slight differences in peak positions and the influence of strain and microstructure on lattice constant estimation. While the diffraction peaks of TiN-3 appear closest to the reference values, the calculated lattice constant (a = 4.262 Å) is slightly larger than that of TiN-2 (a = 4.256 Å). This is likely due to tensile stress or interstitial nitrogen atoms that slightly expand the unit cell, a common phenomenon in sputtered TiN films grown under nitrogen-rich conditions. Structural parameters obtained during XRD analysis are shown in Table 2.

### 3.2. Electrical Measurements of Fabricated MIM Structures

Analysis of the current–voltage characteristics of the fabricated MIM structures revealed that the MIM cells with electrodes having the biggest crystallite sizes (TiN-3 layer) demonstrated the most promising performance as resistive switching devices. These structures were investigated using a broader range of electrical measurements. Structures with smaller crystallite sizes (TiN-2 layer) did not show resistive switching behavior. In this case, current saturation was observed, even at higher voltages. However, structures with the lowest crystallite sizes (TiN-1 layer) manifested forming behavior, which in RRAM devices means a first change from HRS to LRS, but without further resistive switching.

**Figure 5:**
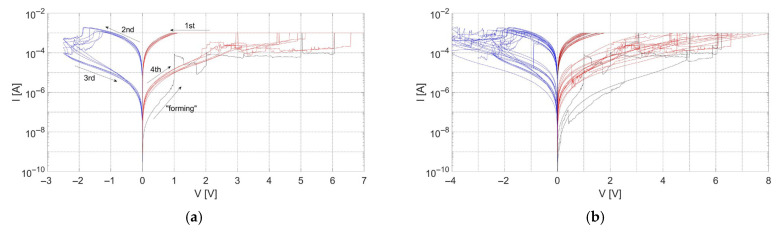
Current–voltage characteristics of structures with TiN-3 layers as electrodes: (**a**) C2C measurement; (**b**) D2D measurement.

Changes in electrical performance during measurements of subsequent cycles of the MIM structure with TiN-3 layers as electrodes are shown in Figure 5a. This type of measurement was named cycle-to-cycle (C2C) and revealed that the memory window (I_LRS_/I_HRS_ at 0.3 V) remained the same in every cycle and was calculated as about 20. Comparison of I-V characteristics of other devices with the same material stack revealed differences in their electrical performance, as shown in Figure 5b. This type of measurement was called device-to-device (D2D) and allowed for the observation of stochastic properties of resistive switching behavior. In this case, a minimal memory window was calculated as one order of magnitude. Despite a few measured cycles, the characteristics shown in Figure 5a,b show the stability of resistive switching in devices with TiN-3 layers as electrodes. Our current study focuses on structural and material-process correlations in the early development phase. Complete endurance testing will be a part of our ongoing work and will be addressed in the future.

As shown in Figure 5b, the current–voltage characteristics exhibit variability between subsequent devices. This variation is primarily observed in the current at the high-resistance state, which depends on the gap between the formed conductive filament and the top electrode. The variability arises from the stochastic nature of ion migration, which affects the shape and size of the conductive filament [24].

**Figure 6:**
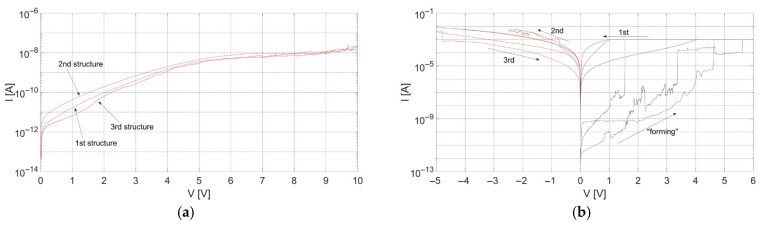
(**a**) Current–voltage characteristics of structures with TiN-2 layer as electrodes; (**b**) Current–voltage characteristics of structures with TiN-1 layer as electrodes.

Structures with TiN-2 layers as electrodes did not show the resistive switching phenomenon. During electrical measurements of these structures, no sudden current changes were observed. A change of current was noticed after increasing the voltage to 14 V throughout the measurement of a few structures. Further analysis of this performance revealed that the current increase was related to the dielectric breakdown of the SiO_x_ layer. In all remaining structures with the TiN-2 films as electrodes, changes in current were observed in the range from 0 V to 5 V. At higher voltages, current saturation was noticed. The described behavior is shown in Figure 6a as an I-V characteristic of an exemplary MIM structure.

Electrical measurements of structures with TiN-1 layers confirmed the possibility of obtaining forming behavior, but further investigations of changing the cell state from LRS to HRS did not bring the desired results. In some experiments, the difference between the low- and high-resistance states was negligible, whereas in others the current increased, indicating that the cell’s resistance decreased. The latter observation is not desirable behavior in RRAM devices. Exemplary current–voltage characteristics of the structures with the electrodes exhibiting the smallest crystallite sizes in the electrode material are shown in Figure 6b.

While crystallite size plays a critical role, it is not the only contributing factor. TiN-1, with the smallest grains and highest full width at half maximum (FWHM) in XRD, likely has high defect density and grain boundary scattering, which disrupts stable filament formation and leads to erratic or unidirectional switching. In contrast, TiN-2, despite moderate crystallinity, may contain intrinsic stress or defect states that promote dielectric breakdown (as observed around 14 V) instead of controlled switching. TiN-3, with the highest crystallinity and more uniform structure, provides a stable medium for repeatable RS behavior.

An additional factor worth considering emerges from the analysis. While both electrodes were deposited using the same conditions, the different substrates (crystalline Si vs. amorphous SiO_2_) can indeed influence the initial nucleation and grain growth. In particular, TiN on Si may promote more ordered growth, while TiN on SiO_2_ may result in slightly different microstructure due to the lack of lattice matching. In this study, it is hard to analyze the upper vs. lower electrodes independently. However, the potential influence of the substrate on microstructure is planned to be explored in future experiments.

**Figure 7:**
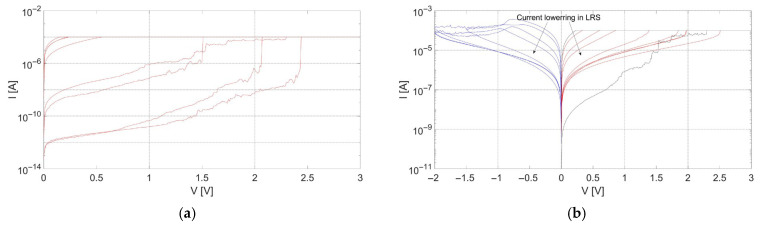
Current–voltage characteristics of structures with extension pads only to the bottom electrodes, presenting (**a**) forming operations; (**b**) C2C measurement.

The performance presented in Figure 5 refers to the structures with extension pads to both the bottom and top electrodes. In the case of devices with the TiN electrodes exhibiting the largest crystallite size, structures with extension pads only to the bottom electrodes were also investigated. During measurements of I-V characteristics, a decrease in forming voltage was noticed, as shown in Figure 7a. The voltage value was lowered from 5.5–6.5 V to 1.5–2.5 V. During the investigation of the structures with extension pads to only the bottom electrodes, current lowering in the low-resistive state throughout measurements of subsequent cycles was noticed, as shown in Figure 7b. This behavior is not commonly observed during the characterization of RRAM devices.

The bottom electrodes of these structures were characterized by the same properties as the electrodes of the devices with extension pads to both BE and TE. The same applies to the dielectric layer and top electrodes. The examined TiN electrodes were characterized by high conductivity (500 kS/m), which excludes that differences in forming voltages could be caused by the series resistance of the extension pad to the top electrode. Conductivity measurements were performed using the typical four-probe method. It can be hypothesized that differences in the electrical performance of structures under investigation are probably caused by mechanical stresses introduced to the structures by placing the electrical probe directly on the top electrode [6].

Intrinsic stress arising during TiN film growth (due to lattice mismatch, grain boundaries, or deposition energy) can significantly affect defect density, grain boundary formation, and consequently the RS behavior. We observed that TiN-1, which exhibits the poorest crystallinity and smallest grain size, also displayed the most erratic electrical behavior, suggesting a high density of internal stress-induced defects and local non-uniformities. However, further analysis of test structures must confirm the latter assumption.

## 4. Conclusions

This study demonstrated the significant impact of stoichiometry and titanium nitride (TiN) electrode crystallinity on the electrical performance of Metal–Insulator–Metal structures exhibiting resistive switching behavior. Adjusting the Ar/N_2_ gas ratio during the reactive magnetron sputtering process affects deposition conditions in the chamber, such as the partial pressure of the sputtering gas. These changes resulted in variations in the Ti/N atomic ratio and film crystallinity, which were systematically achieved and confirmed through spectroscopic ellipsometry, X-ray photoelectron spectroscopy (XPS), and X-ray diffraction (XRD).

A clear trend of increasing titanium content relative to nitrogen was observed with decreasing Ar rate flow, which correlated with a shift in the zero-crossing wavelength of the real part of relative permittivity and was further supported by chemical composition analysis. These changes in composition influenced the crystal structure, particularly crystallite size, and the degree of crystallinity, which affected the MIM devices’ RS properties.

Electrical measurements showed that MIM structures with electrodes composed of highly crystalline TiN exhibited the most promising RS characteristics, including a stable and repeatable memory window and clear cycle-to-cycle performance. Conversely, structures with medium or poorly crystalline TiN electrodes either failed to exhibit RS behavior or required a forming process without further switching, indicating limited or unstable switching capabilities.

Furthermore, mechanical factors such as the placement of electrical probes influenced the observed forming voltages, particularly in structures with top electrodes characterized by large crystallites. A decrease in forming voltage was observed when the top probe was placed directly on the TiN electrode rather than on the extension pad, suggesting a stress-induced effect on switching thresholds.

In conclusion, precise control over the deposition parameters of TiN electrodes, specifically gas flow composition, enables tuning of their physicochemical properties, which directly and critically affect the functionality of RRAM devices. These findings highlight the necessity of optimizing electrode material properties for achieving reliable RS behavior and enhancing device performance and reproducibility in future memory applications.

## Figures and Tables

**Table 1:** Results of XPS measurements.

Sample	Ar/N_2_	Ti [at. %]	N [at. %]	O [at. %]	C [at. %]
TiN-1	50/7	26.1	27.3	22.6	24
TiN-2	40/7	24.6	25.3	24.7	25.3
TiN-3	20/7	23.8	22.8	27.5	25.9

**Table 2:** Calculated data from the XRD measurements.

Sample	a (Å)	D (Å)
Ref.	4.242	-
TiN-1	4.278 ± 0.01	8
TiN-2	4.256 ± 0.079	464.3
TiN-3	4.262 ± 0.01	757.4

## Data Availability

The original contributions presented in this study are included in the article. Further inquiries can be directed to the corresponding author.

## Abbreviations

The following abbreviations are used in this manuscript:

RS	Resistive switching
MIM	Metal–Insulator–Metal
RRAM	Resistive random-access memory
CMOS	Complementary Metal-Oxide-Semiconductor
SRAM	Static random-access memory
DRAM	Dynamic random-access memory
XPS	X-ray photoelectron spectroscopy
XRD	X-ray diffraction
MRAM	Magnetic random-access memory
PCM	Phase-change memory
FeRAM	Ferroelectric random-access memory
HRS	High-resistive state
LRS	Low-resistive state
BEOL	Back-end of line
BE	Bottom electrode
TE	Top electrode
PVD	Physical Vapor Deposition
sccm	standard cubic centimeter
Re(ε)	Real part of relative permittivity
DI	deionized
BE	Binding Energy
C2C	Cycle to cycle
D2D	Device to device
